# Pain and stress assessment after retinopathy of prematurity screening examination: Indirect ophthalmoscopy versus digital retinal imaging

**DOI:** 10.1186/1471-2431-12-132

**Published:** 2012-08-28

**Authors:** M Teresa Moral-Pumarega, Sonia Caserío-Carbonero, Javier De-La-Cruz-Bértolo, Pilar Tejada-Palacios, David Lora-Pablos, Carmen R Pallás-Alonso

**Affiliations:** 1 Department of Neonatology (IMAS12-SAMID), “12 de Octubre”, University Hospital (SERMAS), Madrid, Spain; 2 IMAS12, “12 de Octubre, University Hospital Avenida de Córdoba s/n, 28041, Madrid, Spain

**Keywords:** Diagnostic techniques, Pain measurement, Retinopathy of prematurity, Telemedicine

## Abstract

**Background:**

Increasingly, neonatal clinics seek to minimize painful experiences and stress for premature infants. Fundoscopy performed with a binocular indirect ophthalmoscope is the reference examination technique for screening of retinopathy of prematurity (ROP), and it is associated with pain and stress. Wide-field digital retinal imaging is a recent technique that should be evaluated for minimizing infant pain and stress.

**Methods:**

The purpose of the study was to assess and compare the impact of using a binocular indirect ophthalmoscope (BIO), or wide-field digital retinal imaging (WFDRI) on pain and stress in infants undergoing ROP screening examination. This was a comparative evaluation study of two screening procedures. Ophthalmologic examinations (N = 70) were performed on 24 infants with both BIO and WFDRI. Pain assessments were performed with two specific neonatal scales (Crying, requires oxygen, increased vital signs, expression and sleeplessness, CRIES and, Premature infant pain profile, PIPP) just prior to the examination, and 30 seconds, 1 hour, and 24 hours later after ending the examination.

**Results:**

Changes over time were significantly different between BIO and WFDRI with both scales (PIPP score, *p* = .007, and CRIES score, *p* = .001). Median PIPP score (interquartile interval) at baseline was 4 (3–5). At 30 seconds the score was 8 (6–9) for BIO and 6 (5–7) for WFDRI, respectively. The increase in PIPP score between baseline and 30 seconds was significantly lower with WFDRI (*p* = .006). The median increase in CRIES score from baseline to 30 seconds was 1 point lower for WFDRI than for BIO (*p* < .001). No significant difference in response remained at 1 hour or 24 hour assessments.

**Conclusions:**

A transient short-term pain and stress response occurs with both BIO and WFDRI. Infants examined for screening of ROP with digital retinal imaging present less pain and stress at 30 seconds following completion of the exam when compared with binocular indirect ophthalmoscopy.

## Background

Retinopathy of prematurity (ROP) is a disorder in the development of retinal vessels in premature infants. It is a preventable cause of childhood blindness globally [1]. The efficacy of screening and treatment of ROP depends on early identification of retinal pathology, which is susceptible to treatment [2-4]. Fundoscopy performed by use of a binocular indirect ophthalmoscope (BIO) is the reference examination technique for screening for ROP [2,5]. However, it is associated with pain and stress for premature infants [6,7]. There is substantial evidence to demonstrate that pain relief provided in the intraoperative period decreases infant morbidity [8,9]. This study challenges the current paradigm of pain management in children. Exposure to repeated painful procedures and experiences during the neonatal hospital stay for very premature infants is associated with impaired long-term neurodevelopmental, social, and emotional functions [10-12]. Increasingly, neonatal care units seek to minimize painful experiences and stressful situations for the neonate [13-15]. In the last few years, another retinal examination technique has been developed using wide-field digital retinal imaging (WFDRI) [16,17]. To consider the digital device as an alternative examination to BIO, in addition to comparing its performance during screening, we assessed pain and stress during its application. Recently, less cardiorespiratory stress was observed in children examined with WFDRI than in those subjected to BIO [18-20]. The aim of this study was to compare pain and stress responses measured with specific assessment scales when performing fundoscopy with BIO versus WFDRI.

## Methods

### Patients

This is a comparative evaluation study of a screening procedure performed with collation of prospective data. Criteria for eligibility of participants in the study were the following: 1) compliance with birth weight (≤ 1250 g) or gestational age (≤ 30 weeks) criteria to enter the local screening program for ROP [21]; 2) performing the screening examination during hospital admission; 3) ophthalmologic examination with the two study techniques, BIO and WFDRI. The number of paired examinations required for estimation of a difference between the techniques at 1 point on the pain scale with a confidence level of 95% and a power of 80%, was 30. Approval from the Hospital Ethics Committee and written informed consents were obtained from the parents of the patients.

### Ophthalmologic examination procedures

For all participants, each examination was performed by the same ophthalmologist (PTP), with a BIO and with a WFDRI, using the Retcam 120 camera-9 system (RetCam-II, Clarity Medical Systems, Pleasanton, CA). In all cases, examination was initially performed with Retcam, and after an interval of 3 to 5 days, with a BIO. The first screening examination was generally performed between 6–8 postnatal weeks. To dilate the pupils, one drop of 0.5% cyclopentolate, and one drop of 2.5% phenylephrine was administered in each eye, and the dose was repeated after 30 minutes. Several minutes later and 2 minutes prior to performing the examinations with both techniques, we administered 24% oral sucrose at 0.2 cc per os (p.o.) and by sucking with a pacifier. The local anesthetic [oxybuprocaine and tetracaine (1 mL each), 1 mg of tetracaine chlorohydrate, and 4 mg of oxibuprocaine chlorohydrate] were applied topically, and an eyelid speculum was used (premature blefarostate with a 16 mm opening and 4 cm in length). Standard BIO examination was performed with scleral indentation, and Retcam examinations were performed without scleral indentation. For WFDRI examination, a Viscotears R gel was applied for contact between the camera lens and the eye. During examinations or during the period between examinations, infants did not receive other analgesics or sedatives as part of their normal treatment.

### Patient assessments

Pain and stress assessment was performed with two scales validated for neonatal use [22,23]. The CRIES scale has a value range of 0 to 10. The PIPP scale has a value range of 0 to 21. Both scales assess physiological and behavioral parameters, but the PIPP also includes information on gestational age at birth. Blood pressure was measured non-invasively (Dinamap; GE Healthcare, Chalfont, St. Giles, UK). Heart rate (HR) and oxygen saturation (SaO_2_) were collated with a pulse-oximeter (Nellcor; Covidien, Dublin, IR). Respiratory frequency (RF) was recorded manually. The time sequence of assessments was as follows: baseline (prior to administration of the cycloplegic), 30 seconds following completion of the examination, at 1 hour, and at 24 hours. During the examination the nurse in charge of the patient helped with containment and swaddling, while the pediatric ophthalmologist performed the examination and a neonatologist (MMP or SCC) performed and reported the pain measurements. In our study setting, it was not feasible to rate the parameters of the scales during the exam but only after the end of it. Facial expression could not be assessed while speculum was still in the eye or while WFDRI was being used. The first assessment following the exam was performed 30 seconds after the end of the exams. Prior to commencing the study, both neonatologists were trained specifically on assessment of pain scales until a reliable interobserver agreement was attained. During pilot studies on 10 exams, no disagreement was observed between both assessors. No video recording was performed.

At the time of the examination, no infant had a diagnosis of sepsis or hypovolemia, which could interfere with the assessment of cardiorespiratory indices. Corrected age, days of life, location of the infant (intensive care or intermediate care), respiratory care required (mechanical ventilation, CPAP, oxygen in a blender with nasal cannula, and without additional oxygen), poor saturation, whether any recommended analgesic was administered, and the duration of the examination, were also collated.

### Data analysis

Measurable and categorical variables are expressed as mean (SD, standard deviation) or median (II, interquartile interval) and range, or frequency distribution (95% confidence intervals), respectively. Analysis of variance for repeated measurements was used to test for statistical significance of difference over time between examination techniques. Differences between 30 seconds, 1 hour, and 24 hours following examination and baseline pain assessments were computed for each pair of examinations, then were compared and further stratified by additional relevant determinants. All statistical comparisons used paired t-tests, based on within examination differences. The data analysis was generated using SAS software (SAS Institute Inc, Cary, NC, USA).

## Results

### Patient data

**Figure 1:**
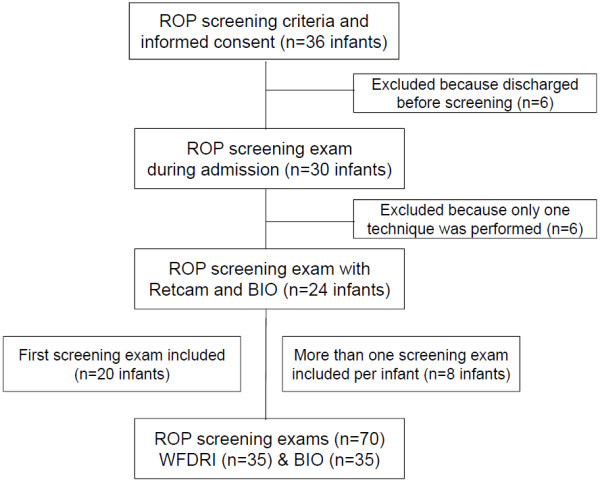
**Flow diagram of the study participants.** Abbreviations: ROP, Retinopathy of prematurity; WFDRI, wide-field digital retinal imaging; BIO, binocular indirect ophthalmoscope.

During the 6 month recruitment period between November 2007 and May 2008, 36 infants complied with the screening program’s admission criteria. Twelve infants were excluded (Figure 1). Finally, 24 infants underwent 70 examinations in total, 35 with Retcam and 35 with BIO. Mean gestational age of the 24 infants eligible for the final analysis was 27 weeks (SD 1.8), with a range of 24 to 30 weeks. Mean weight of the neonates were 895 g (171) with a range of 585 g to 1250 g. Eleven infants were males (46%). Mean postnatal age at the time of the initial examination was 6.4 (0.97) weeks, with a range of 4.7 to 8.2 weeks. Of the 70 examinations performed, 33 (47%) occurred in the neonatal intensive care unit. In 45 (64.3%) of the examinations, patients did not receive respiratory care.

### Change over time in primary pain outcome measures

**Figure 2:**
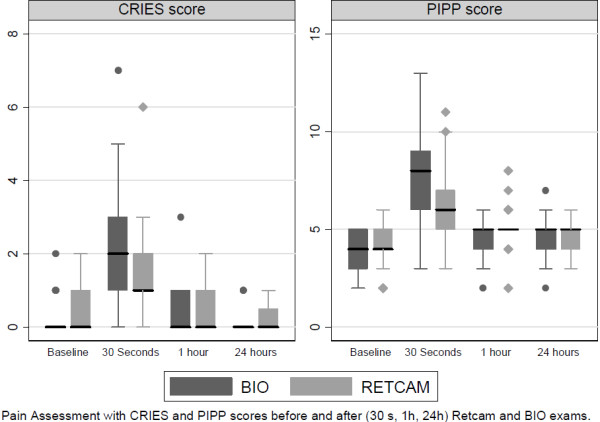
**Pain Assessment with CRIES and PIPP scores before (baseline) and after (30 seconds, 1 hour, and 24 hours) WFDRI and BIO exams.** Abbreviations: WFDRI, wide-field digital retinal imaging; BIO, binocular indirect ophthalmoscopy.

The progression of pain and stress assessment values over time following fundoscopy differed depending on the ophthalmologic technique, both with the CRIES scale, *p* = .002, and with the PIPP scale, *p* = 0.007. With both examination techniques and assessment scales, there was a significant increase in scores at 30 seconds followed by a return to baseline values (Figure 2). Using the CRIES scale, the 30 second mean increase was 1.2 after WFDRI and 2.3 after BIO (*p* = .000). Using the PIPP score, the 30 second mean increase was 2.1 after WFDRI and 3.4 after BIO (*p* = .006). No difference from baseline was observed with any pain assessment scale at 1 hour and 24 hours (Table 1). Desaturation was observed in exactly the same proportion, 8.6% (3/35) of examinations, with WFDRI and with BIO.

**Table 1:** Pain assessed with CRIES and PIPP scores 30 seconds following ROP screening examination with WFDRI (n = 35) and BIO (n = 35)

**Time**	**CRIES score**			**PIPP score**		
	**WFDRI**	**BIO**	**Difference in pain score (BIO-WFDRI) from baseline**	**WFDRI**	**BIO**	**Difference in pain score (BIO-WFDRI) from baseline:**
	**Mean (SD)**	**Mean (SD)**	***p *value**	**Mean (SD)**	**Mean (SD)**	***p *value**
Baseline	0.4 (0.7)	0.2 (0.5)	-	4.1 (1.1)	4.0 (1.0)	-
30s after	1.6 (1.1)	2.5 (1.4)	0.000	6.2 (1.9)	7.4 (2.3)	0.006
1 h after	0.4 (0.6)	0.3 (0.6)	0.79	5.1 (1.2)	4.7 (1.0)	0.16
24 h after	0.3 (0.4)	0.1 (0.4)	0.62	4.7 (0.7)	4.7 (0.9)	0.75

Abbreviations: CRIES score, Crying, Requires oxygen, Increased vital signs, Expression and Sleeplessness; PIPP score, Premature Infant Pain Profile; ROP, retinopathy of prematurity; WFDRI, wide-field digital imaging; BIO, Binocular indirect ophthalmoscopy; SD, standard deviation; s = seconds; h = hours.

### Determinants of stress response

The effects of patient characteristics on the response to pain and stress were analyzed based on gestational age (≤ 26 weeks or > 26 weeks), location at the time of examination (intensive or intermediate care), type of respiratory care at the time of examination (nasal continuous positive airway pressure CPAP or intubated and connected to mechanical ventilation compared with nasal oxygen or nothing), and whether it was the initial or a subsequent screening examination (Table 2). No determinant showed significant differences (all *p* values > .2, data not shown) in 30 second baseline assessments for both techniques and both pain scales. It is notable that in relation to gestational age, the difference (BIO-WFDRI) in pain assessed at 30 seconds with CRIES was significantly higher in the >26 weeks group compared with the more immature infant group (≤ 26 weeks) (*p* = .03).

**Table 2:** Increase in pain score values (CRIES and PIPP) 30 seconds after ROP screening examination: Stratified comparison of the difference between techniques

**Determinant**	**Increase in pain score from baseline to 30 seconds following examination**					
	**CRIES score**			**PIPP score**		
	**WFDRI**	**BIO**	**Difference between strata**	**WFDRI**	**BIO**	**Difference between strata**
	**Mean(SD)**	**Mean(SD)**	**(*p *value)**	**Mean (SD)**	**Mean (SD)**	**(*p *value)**
Gestational age:						
- ≤ 26 weeks (n=18)	1.56 (1.3)	2.06 (1.7)	0.03*	2.28 (1.9)	3.28 (2.1)	0.51
- > 26 weeks (n=17)	0.88 (1.1)	2.53 (1.0)		1.88 (1.9)	3.47 (2.2)	
Location:						
- NICU (n=14)	0.79 (1.1)	2.07 (1.7)	0.52	2.14 (1.6)	4.00 (2.2)	0.29
- Intermediate (n=21)	1.52 (1.3)	2.43 (1.2)		2.05 (2.1)	2.95 (2.1)	
Order of screening exam:						
- 1^st^ exam (n=20)	1.00 (0.9)	2.20 (1.6)	0.56	2.05 (1.9)	3.70 (2.0)	0.35
- >1^st^ exam (n=15)	1.53 (1.6)	2.40 (1.1)		2.13 (2.0)	2.93 (2.5)	
Respiratory support						
- no/n. cannula (n=31)	1.32 (1.2)	2.19 (1.3)	0.06	2.06 (1.9)	3.16 (2.1)	0.24
- CPAP or ET (n=4)	0.50 (1.2)	3.00 (2.1)		2.25 (2.2)	5.00 (2.1)	

Abbreviations: CRIES score, Crying, Requires oxygen, Increased vital signs, Expression and Sleeplessness; PIPP score, Premature Infant Pain Profile; ROP, retinopathy of prematurity; WFDRI, wide-field digital imaging; BIO, Binocular indirect ophthalmoscopy; SD, standard deviation; ET, Endotraqueal tube; CPAP, Continuous positive airway pressure.

### Examination time

The mean duration for examination was 3.72 minutes (SD 2.3), with a range of 2 to 11 minutes with BIO, and 3.70 minutes (2.17), with a range of 1 to 12 minutes with WFDRI.

## Discussion

The results of this study showed that there is less pain and stress following completion of the exam in premature children when screening for ROP is performed with WFDRI compared with examination with BIO. Several studies have already shown that examination with BIO produces pain in premature infants [6,7,24]. Few reports compared the effects produced by both examination techniques (WFDRI/BIO) [18-20].

Mukherjee *et al*. [18], in a population of premature infants with similar weight and gestational age to that of our study, also encountered less signs of stress/pain when the examination was performed with WFDRI. The examination with BIO was performed on some infants and with WFDRI on others. In our study, use of both examination techniques was an eligibility criterion that provided a more robust comparison. In the previous report [18], the main outcome measure was variation in cardiorespiratory parameters. In addition to cardiorespiratory variables (Heart rate, blood pressure, SaO_2_), we used specific pain scales validated for the neonatal setting that included facial expression, crying, sleeplessness, and gestational age, which provided a more comprehensive assessment of the pain and stress experience in neonates. Baseline assessment values were taken prior to administering the drops so as not to interfere with the possible reported side effects [24]. Using the assessment of CRIES and PIPP scales in our study setting, it was not feasible to obtain the parameters of the scales during the exam, but only after the end of the exam. Facial expression could not be assessed while speculum was still in the eye or while WFDRI was being used. We decided to homogenously assess these parameters at 30 seconds after the end of all exams. The assessment at 24 hours after the procedure was included because higher values were previously reported in extremely premature infants [25].

Mehta *et al*. [20] compared WFDRI with speculum and BIO with and without speculum in a series that included 12 infants. These authors found no differences in cardiorespiratory indices and facial assessment between WFDRI and BIO examinations with speculum. They showed less pain with BIO when the examination was performed without speculum, and concluded that it might be appropriate not to use a speculum in particularly ill infants. In our study, the standard technique with eyelid speculum was used, with WFDRI and BIO, to allow for full visualization of the retina and for valid comparisons. Rush *et al.*[7] and Laws *et al*. [24] concluded that adrenergic manifestations of stress and pain, and modifications in SaO_2_ following examination with BIO appeared after handling of the eye and use of the speculum. The only effect which could be attributed to the mydriatic drug used was increase in mean blood pressure.

Recently, Dhaliwal *et al*. [19] assessed PIPP scores recorded in the first minute of examination with WFDRI or BIO in 76 un-swaddled, non-nested infants, with no use of pacifier or oral sucrose. Exceedingly high pain score values at baseline and no differences between techniques were observed. Current recommendations [2] and recent evidence [15] should minimize the adverse effects of examinations in the future.

Regarding time employed during the examinations procedure, no differences were found between the two techniques. In this study, WFDRI preparation time was not included, only examination time. Mukherjee *et al*. [18] observed longer examination times with WFDRI when including the time to set up the instrument.

Pain is a multifactorial phenomenon with physiological and behavioral aspects, modified in infants by factors such as gestational age, state of health (sepsis and hypovolemia may lead to tachycardia and changes in blood pressure), and maturity [23]. In our study, none of the determinants analyzed showed significant differences in pain measurements for both techniques and both pain scales. Rush *et al*. [7] and Mukherjee *et al*. [18] did not report higher systemic manifestations or pain in responses to mydriasis and fundoscopy in lower gestational age infants. The significantly higher difference observed in CRIES score between BIO and WFDRI, in the gestational age group >26 weeks might be explained by higher baseline pain values in more immature infants. Pain and stress responses for successive examinations did not increase when compared with the first examination, with intermediate care *vs.* NICU, or with higher respiratory support *vs.* none. These determinants were not considered in the aforementioned studies [18-20].

A potential drawback to this study is that, per protocol the technique with WFDRI was performed first, followed by the BIO technique after 3 to 5 days. The time interval between both examinations was sufficiently long enough to minimize any interference between them. Similar baseline pain assessment values were observed before WFDRI and BIO examinations. The two scales used in our study for outcome assessment provide more consistency to the results of the study.

The fact that scleral indentation with the WFDRI was not necessary to visualize the retina is of paramount importance because this factor is significantly related to pain and stress. The lower light intensity used with WFDRI might cause less photophobia and less discomfort for the infant.

## Conclusions

In addition to producing less pain and stress during fundoscopy of premature infants, WFDRI is a technique which enables reviewing images once the procedure has been completed because programs for telemedical diagnosis of ROP have been developed.

This study confirms that infants undergoing fundoscopy for ROP show a transient short-term pain and stress response with both BIO and WFDRI. However, when ophthalmologic examination for screening of retinopathy of prematurity is performed with digital retinal imaging, infants present less pain and stress when compared with those examined with a binocular indirect ophthalmoscope. Ophthalmology examinations are repeated during admission. Therefore, examination with WFDRI could reduce painful and stressful experiences to which premature infants are exposed. Strategies to minimize the impact of adverse experiences and environmental factors in premature infants will contribute to optimal neurodevelopment of preterm neonates.

## Endnotes

Pain and stress of premature infants undergoing screening for retinopathy of prematurity should be minimized. An increased pain and stress response occurs with wide-field digital retinal imaging (WFDRI) and with the binocular indirect ophthalmoscope (BIO) when both techniques are applicable. Less transient short-term pain and stress responses are observed with WFDRI when compared with BIO.

## Abbreviations

BIO: Binocular indirect ophthalmoscopy; CPAP: Continuous positive airway pressure; CRIES: Crying, requires oxygen, increased vital signs, expression and sleeplessness; NIDCAP: Newborn individualized developmental care and assessment program; PIPP: Premature infant pain profile; WFDRI: Wide-field digital retina imaging.

## Competing interests

All authors declare they have no competing interests.

This work was partially supported by an unrestricted research grant (2006/099) from Fundación Mutua Madrileña, Spain. The funding organization played no role in the design or conduct of this research.

## Author contributions

MM made substantial contributions to concepts and design, acquisition of data, analysis and interpretation of data, in drafting the manuscript and revision for important intellectual content, and has given final approval of the published version. SC made substantial contributions to concepts, design, and acquisition of data. JDC made substantial contributions to concepts and design, analysis, interpretation of data, drafting the manuscript, revising it critically for important intellectual content, and gave final approval of the published version. PT made substantial contributions to concepts and design, performed the screening examination with indirect ophthalmoscopy, and did the digital retinal imaging. PT also helped revise the manuscript for important intellectual content and gave final approval of the published version. DL made substantial contributions to concepts and design, analysis, and interpretation of data. CP made substantial contributions to concepts and design, was involved in drafting the manuscript, helped to revise it critically for important intellectual content, and gave final approval of the version to be published.

## Pre-publication history

The pre-publication history for this paper can be accessed here:

http://www.biomedcentral.com/1471-2431/12/132/prepub
